# Physical activity particpation and cardiovascular fitness in people leaving with HIV. A one-year longitudinal study

**DOI:** 10.1186/1742-4690-9-S1-P63

**Published:** 2012-05-25

**Authors:** S Fillipas, FM Cicuttini, AE Holland, CL Cherry

**Affiliations:** 1 Physiotherapist at the Alfred, Melbourne, Australia

## Introduction

Physical activity (PA) and cardiovascular fitness (CVF) are beneficial for HIV-infected individuals, however long-term effects are unknown. This study aimed to document long-term habitual PA and CVF in stable, HAART-treated individuals with HIV, explore relationships to body composition, body image and cardiovascular disease (CVD) risk and evaluate PA determinants.

## Materials and methods

This was a 12-month prospective, longitudinal cohort study. Eighty ambulant HIV-infected adults (aged ≥18 years) on HAART were recruited from The Alfred Hospital Infectious Diseases clinic and local HIV community clinics. PA was reported using the International Physical Activity Questionnaire and CVF assessed using the Kasch Pulse Recovery Test.

## Results

19-37% participants reported suboptimal PA levels at each study visit, while PA and CVF were largely stable over the study period. Higher CVF was associated with better body composition and this association persisted over time (p=0.05). Greater total energy expenditure was associated with improved body image (r=-0.325, p=0.027) but notCVD risk. At baseline, the proportion of subjects in a permanent relationship was higher among active versus inactive participants [47.7% versus 13.3% (p=0.032)]. A similar trend was seen at six and 12 months.

## Conclusions

In this stable, HAART-treated HIV–infected cohort a sub-optimal level of PA participation was observed. CVF was associated with improved body composition, suggesting HIV- infected individuals should be encouraged to improve and maintain CVF. Increasing PA levels were associated with improved perceived body image, supporting use of PA to improve this aspect of psychological well being. Being in a permanent relationship was associated with higher PA levels suggesting that social isolation may be a risk factor for inactivity in those with HIV. Further work, including larger cohorts and longer follow up is needed to explore factors that influence PA and CVF in HIV. This study found benefits for both long-term PA and CVF for chronic HIV-infection however intervention studies are required to define the benefits obtainable for improving long-term PA uptake and CVF in this population.

